# Preliminary Validation of Japanese Version of the Parental Burnout Inventory and Its Relationship With Perfectionism

**DOI:** 10.3389/fpsyg.2018.00970

**Published:** 2018-06-20

**Authors:** Taishi Kawamoto, Kaichiro Furutani, Maryam Alimardani

**Affiliations:** ^1^Department of Psychology, Chubu University, Kasugai, Japan; ^2^Faculty of Business Administration, Hokkai-Gakuen University, Sapporo, Japan; ^3^Department of Cognitive Science and Artificial Intelligence, Tilburg University, Tilburg, Netherlands

**Keywords:** parental burnout, job burnout, perfectionism, personal standards, concern over mistakes, Japan

## Abstract

Parenting is a precious experience and also a very hard task, which could result in *parental burnout* for some parents. The present study sought to validate a Japanese version of the Parental Burnout Inventory (PBI-J) by replicating and extending the pioneering work of Roskam et al. (2017). We conducted a web survey (*N* = 1200) to first validate the PBI-J and second to investigate the association between the PBI-J and perfectionism as a new interrelation. Similar to the prior study of Roskam et al. (2017), confirmatory factor analysis supported a model of three-factor structure of the PBI-J: emotional exhaustion, lack of personal accomplishment, and emotional distancing. In addition, we found low to moderate correlations of parental burnout with job burnout, parental stress, and depression. These findings provided initial evidence for validity of the PBI-J and suggested that parental burnout appeared to be different from job burnout. Our further evaluation of perfectionism confirmed such a difference between parental and job burnout by showing that parental perfectionism [i.e., combination of parental personal standards (PS) and parental concern over mistakes (CM)] has a unique contribution to parental burnout than does job perfectionism (i.e., combination of job PS and job CM). In addition, CM was positively correlated with burnout in both domains whereas the associations between PS and burnout were more complex. Finally, the proportion of parents experiencing burnout was estimated to lie somewhere between 4.2 and 17.3% in Japan. Overall, the present study confirmed preliminary validity of the PBI-J and found that parental perfectionism is one of the vulnerability factors in parental burnout.

## Introduction

Parents often struggle with parenting on a daily basis and some of them get physically and emotionally exhausted. Parenting is a precious experience and one of the most difficult and complex tasks, as there appears to be no explicitly correct answer to the question, “What is the best parenting style?” Some parents try their best to perfectly nurture their children by having high standards and avoiding mistakes in parenting. Such efforts, however, may result in parental burnout—emotional disorder related to the context of parenthood (Roskam et al., 2017). One previous study revealed that 2–12% of parents might experience parental burnout in Europe (Roskam et al., 2017). Other studies have indicated that parental burnout was predicted by individual differences, such as neuroticism (Le Vigouroux et al., 2017; Mikolajczak et al., 2018). Although these seminal studies shed light on the importance of focusing on parental burnout, this research topic is still in its infancy, and needs further investigation.

The present study sought to further improve our understanding of parental burnout in two ways. First, we sought to validate a Japanese version of the Parental Burnout Inventory (PBI-J) by replicating the pioneering work of Roskam et al. (2017). Measurement validation is the first step in understanding whether parental burnout is also prevalent outside of Europe or merely a culturally specific phenomenon. We translated the original PBI into Japanese, and tested the construct validity of the PBI-J through confirmatory factor analysis (CFA) and correlational analysis. Second, we focused on perfectionism as a new vulnerability factor of parental burnout. Although previous studies have revealed that perfectionism is associated with burnout in job, sport, and education domains (e.g., Hill and Curran, 2016), whether it is the same for parenting domain remains unclear. We sought to extend previous research by investigating the associations between perfectionism and burnout in both parenting and job domains.

### Parental Burnout

Parental burnout encompasses three dimensions: emotional exhaustion, emotional distancing, and lack of personal accomplishment (Roskam et al., 2017). Emotional exhaustion captures aspects reflecting an overwhelming exhaustion related to one’s parental role; emotional distancing captures aspects reflecting the tendency to distance oneself from one’s children; and lack of accomplishment captures aspects reflecting a sense of ineffectiveness in one’s parental role (Roskam et al., 2017; Mikolajczak et al., 2018). This three-factor structure corresponds to that of job burnout: emotional exhaustion, depersonalization, and lack of personal accomplishment (Maslach and Jackson, 1981). Depersonalization became emotional distancing in the PBI because parents do not really dehumanize their own children (Roskam et al., 2017).

Although some previous studies have already investigated burnout in the parenting domain (e.g., Norberg, 2007, 2010; Lindström et al., 2010, 2011; Lindahl Norberg et al., 2014), Roskam et al. (2017) significantly improved our understanding of parental burnout by developing the Parental Burnout Inventory (PBI). They developed and validated the PBI by focusing on proximity to and distinctiveness from job burnout. They found the evidence of proximity to job burnout: the PBI encompassed three dimensions—emotional exhaustion, emotional distancing, and lack of personal accomplishment. They also provided preliminary evidence for distinctiveness from job burnout: the PBI had low to moderate correlations with job burnout. The PBI also had low to moderate correlations with depression and child-rearing stress, indicating that parental burnout is not simply job burnout, depression, or child-rearing stress. The first purpose of the current study was to validate a Japanese version of the PBI by investigating whether the aforementioned findings—proximity to and distinctiveness from job burnout—would also be found in Japan.

The PBI could estimate the prevalence of parental burnout using the cutoff points (e.g., above 67 or 88, Roskam et al., 2017; Mikolajczak et al., 2018). These cutoff points were based on 1.5 standard deviation over the mean of the samples (i.e., above 67) or parents who experienced each item at least once a week (i.e., above 88). Using these cutoff points, Roskam et al. (2017) found that 1.3–8.8% of parents would be considered to be experiencing parental burnout in Europe. Following their work, we sought to investigate the prevalence of parental burnout in Japan using two criteria: the PBI-J scores above 67 and 88.

After the pioneering work of Roskam et al. (2017), some other studies sought to improve our understanding of parental burnout by investigating the antecedents or risk factors of parental burnout (Le Vigouroux et al., 2017; Mikolajczak et al., 2018). For example, Le Vigouroux et al. (2017) found that high neuroticism, low agreeableness, and low conscientiousness were possible risk factors for experiencing parental burnout. Mikolajczak et al. (2018) revealed that stable traits of a parent such as emotional stability and emotional intelligence explained larger variances in parental burnout (i.e., 22%) than did sociodemographic variables, which explained a notably low variance (i.e., 3%). The second purpose of the current study was to examine a number of risk factors of parental burnout in Japan. Similar to a previous study (Mikolajczak et al., 2018), we measured sociodemographic variables (e.g., number of children, household income, education level, and work hours per weeks) and examined whether sociodemographic variables have some effect on parental burnout in Japan as well. As a new risk factor of parental burnout, we focused on perfectionism in terms of a stable trait, which has been shown previously as one of the risk factors of burnout in job, sport, and education domains (e.g., Hill and Curran, 2016).

### Association Between Parental Perfectionism and Parental Burnout

Perfectionism encompasses two superordinate dimensions: perfectionistic strivings and perfectionistic concerns (e.g., Frost et al., 1993; Stoeber and Otto, 2006; Stoeber and Gaudreau, 2017). Perfectionistic strivings capture aspects reflecting extremely high standards for performance and a self-oriented striving for perfect results. In contrast, perfectionistic concerns capture aspects such as concerns about making mistakes, doubts about actions, feelings of discrepancy between one’s standards and performance, and fears of negative evaluation by others if one fails to be perfect (e.g., Stoeber and Otto, 2006; Stoeber and Gaudreau, 2017).

Previous studies have revealed that perfectionism is associated with burnout in several domains including job, sports, and education (e.g., Zhang et al., 2007; Stoeber and Rennert, 2008; Hill and Curran, 2016). Generally, perfectionistic strivings were unrelated or negatively related to burnout, whereas perfectionistic concerns have been consistently positively associated with burnout symptoms (e.g., Zhang et al., 2007; Hill et al., 2008; Stoeber and Rennert, 2008; Shih, 2012; Caliskan et al., 2014; Hill and Curran, 2016). Thus, overall perfectionism (the combined effect of perfectionistic strivings and concerns) tends to lead individuals to increased burnout (e.g., Stoeber and Damian, 2016). Although evidence of the relationship between the perfectionism and burnout has been gathered across multiple domains, to date there has been no study that investigated the association between parental perfectionism (e.g., high standards and concern over mistakes (CM) in parenting domain) and parental burnout. We therefore investigated the association between perfectionism and burnout in the parenting domain to further understand the perfectionism-burnout link. We predicted that parental perfectionistic strivings (e.g., high standards in parenting) would be unrelated or negatively correlated with parental burnout. In contrast, parental perfectionistic concerns (e.g., CM in parenting) would be positively correlated with parental burnout. Finally, overall parental perfectionism would be positively correlated with parental burnout.

In addition to the effect of perfectionism *dimensions* (e.g., perfectionistic strivings and concerns) on burnout, it is conceived that *domain specificity* of perfectionism (e.g., job and parenting) also has a significant influence on the associations between perfectionism and outcome variables (e.g., Dunn et al., 2011; Stoeber and Yang, 2015). For example, previous studies have revealed that domain-specific perfectionism (e.g., sport or physical appearance perfectionism) often explains variance in domain-specific outcome variables (e.g., body image or eating disorders) above and beyond general perfectionism measures (e.g., Dunn et al., 2011; Stoeber and Yang, 2015). We therefore measured both parental and job perfectionism to investigate the association between perfectionism and burnout in detail. We predicted that parental perfectionism would have a unique and more distinctive contribution to parental burnout than would job perfectionism. More specifically, we hypothesized that the association of parental burnout would be stronger with parental perfectionism than job perfectionism.

### The Current Study

We sought to validate a Japanese version of the PBI by testing whether the PBI-J encompasses three dimensions—emotional exhaustion, emotional distancing, and lack of personal accomplishment—as well as investigating the associations between these three dimensions and job burnout, depression, and child-rearing stress. In addition, we sought to understand the prevalence of parental burnout in Japan by using cutoff points (i.e., 67 points above or 88 points above). Finally, we investigated the risk factors of parental burnout by focusing on sociodemographic variables and perfectionism. We measured personal standards (PS) and concern over mistakes (CM) as indicators of perfectionistic strivings and concerns, respectively (e.g., Stoeber and Gaudreau, 2017; Kawamoto and Furutani, 2018).

## Materials and Methods

### Participants

Through a web survey, 1,200 working parents who live with at least one child participated in the present study (600 mothers, *M*_age_ = 44.1, Range = 24–65 years, *SD* = 7.5). Participants gave written informed consent through the web survey. They were recruited by a pooling company (Rakuten Research). The Human Research Ethics Committee of the University of Tokyo approved the study protocol. **Table 1** summarizes the characteristics of participants in the current study.

**Table 1:** Characteristics of participants in the current study.

	*N* or *M* [range]
Children’s age	[0–38]
Number of children	*M* = 1.79 [1–5]
**Marital status**	
Living with a partner	1079 (89.92%)
Single parent	121 (10.08%)
**Education level^a^**	
9 years (junior high school)	20 (1.68%)
12 years (high school)	271 (22.73%)
12 to 16 years (technical or 2-year college)	316 (26.51%)
16 years (undergraduates)	504 (42.28%)
>16 years	81 (6.80%)
**Work-related variables**	
Annual net income^b,c^	*M* = 6.78 (*SD* = 2.99)
Working part-time	347 (28.92%)
Working hours per week	34.71 h (*SD* = 19.30)


*N = 1200 except for ^*a*^N = 1192, ^*b*^N = 1103, ^*c*^6 = 5 million to 6 million yen, 7 = 6 million to 7 million yen.*

### Measures

#### Demographic Variables

We measured several demographic variables similar to those in previous studies (Roskam et al., 2017; Mikolajczak et al., 2018). Participants were asked about their gender, age, number of children, age of children, type of family (single parent or not), level of education (junior high, high school, technical or 2-year college, university, or graduate school), annual net income (1 = less than 1 million yen, 2 = 1 million to 2 million yen, …, 10 = 9 million yen to 10 million yen, 11 = over 10 million), forms of work (full-time or part-time), and working hours per week. For the level of education and annual net income, some participants did not give an answer.

#### Parental Burnout

Parental burnout was measured using the PBI (Roskam et al., 2017). The PBI included eight items measuring emotional exhaustion, eight measuring emotional distancing, and six measuring personal accomplishment. Participants rated each item using a 7-point scale ranging from 0 (Not at all) to 6 (Every day). The original PBI was translated into Japanese and back-translated by a translation agency (Crimson Interactive Inc., Japan) to ensure the quality and accuracy of the content. Permission to use and translate the PBI was acquired from Dr. Roskam, who developed the original version. Additionally, permission was acquired from Mind Garden Inc., which holds the copyright to the PBI. The translated and back-translated questionnaires were compared and revised by an English–Japanese bilingual researcher, where special attention was given to preserve the semantic and conceptual equivalence of the original version. Next, Dr. Roskam confirmed that there were no semantic and conceptual differences between the original questionnaire and the back-translated version.

#### Job Burnout

We used the Japanese Burnout Inventory (JBI: Tao and Kubo, 1996; Kubo, 1998), which was widely used in Japan. The JBI included seven items measuring emotional exhaustion, seven measuring depersonalization, and six measuring personal accomplishment. The JBI used completely different items from those of Maslach Burnout Inventory (MBI), which was often used in burnout research outside of Japan. However, the factor constructs of the JBI were similar to those of the MBI, and its reliability has been validated in previous studies (e.g., Kubo, 2014). Participants rated each item using a 7-point scale ranging from 0 (Not at all) to 6 (Every day).

#### Parental and Job Perfectionism

We used the Japanese version of the Multidimensional Perfectionism Scale (J-MPS, Sakurai and Ohtani, 1997), which was developed based on the Frost Multidimensional Perfectionism Scale (Frost et al., 1990) to measure perfectionistic dimensions in Japanese participants. The J-MPS includes four items measuring CM and five measuring PS (see Kawamoto and Furutani, 2018 for details of items).

To measure parental and job perfectionism separately, we used different instructions and opening phrases as was done in previous studies (e.g., Mitchelson, 2009; Dunn et al., 2011; Deuling and Burns, 2017). For parental perfectionism, we instructed participants that the items described “parenting,” and each item started with the phrase “Regarding parenting.” For job perfectionism, we instructed them that the items described “job,” and each item started with the phrase “Regarding job.” Participants rated each item on a 6-point scale ranging from 1 (Not at all) to 6 (Very much).

#### Child-Rearing Stress

Child-rearing stress was measured using the Child-Rearing Stress Scale (Miyata, 2002). Participants rated how often they experienced stressors in six situations (e.g., I feel irritated about myself for not enjoying rearing my children), on a scale from 1 (Not at all) to 4 (Very often). A previous study supported the reliability and validity of this scale (e.g., Miyata, 2002; Kawamoto and Furutani, 2018).

#### Depression

Depression was measured using the Todai Health Index Depression Scale (Aoki et al., 1974). Participants rated their feelings on a 3-point scale that included 1 (No), 2 (neither Yes or No) and 3 (Yes). Previous studies supported the reliability (e.g., α = 0.91: Kawamoto et al., 2017) and validity of this scale (Kawada et al., 1999).

### Data Analysis

We conducted a CFA on the PBI-J using AMOS 23.0. The measurement model included the three latent variables representing the concepts of emotional exhaustion, emotional distancing, and lack of personal accomplishment. Their indicators consisted of eight items for emotional exhaustion, eight for emotional distancing, and six for lack of personal accomplishment. Analyses were conducted based on the covariance matrix using maximum likelihood estimation. The comparative fit index (CFI) (Marsh et al., 1988) and the root mean-square error of approximation (RMSEA) (Byrne, 2001) were used to evaluate the model fit similar to a previous study (Roskam et al., 2017). CFIs close to 0.90 or larger are acceptable, while values higher than 0.95 indicate a better fit to the data. RMSEA under 0.08 are acceptable, but values less than or equal to 0.06 are preferable (Hu and Bentler, 1999).

The associations between the PBI-J and perfectionism were examined in several ways. First, we investigated correlations among parental perfectionism, parental burnout, job perfectionism, and job burnout. Next, to test the influence of domain specificity of perfectionism, we conducted structural equation modeling using AMOS 23.0. The model included two high-order latent variables representing the concepts of parental and job perfectionism, and two latent variables representing the concepts of parental and job burnout. Next, to test the influence of perfectionism dimensions as well as sociodemographic variables on parental burnout, we conducted multiple regression analysis. Sociodemographic variables were entered into Step 1 and we added parental PS, parental CM, job PS, and job CM into Step 2.

Finally, we estimated the prevalence of parental burnout using two cutoff points: PBI scores above 67 and 88.

## Results

### Descriptive Statistics, CFA, and Reliability of PBI-J

**Table 2** shows standardized regression weights from CFA, mean, standard deviation, skewness, and kurtosis for each item of the PBI-J. CFA revealed that fit index of three-factor model of the PBI-J was acceptable [CFI = 0.90, RMSEA = 0.07, χ^2^(206) = 1533.22, *p* < 0.001]. The standardized factor loadings ranged between 0.45 and 0.83. Correlations between emotional exhaustion and emotional distancing, emotional exhaustion and lack of parental accomplishment, and emotional distancing and lack of parental accomplishment factors were 0.80, 0.08, and 0.05, respectively. Cronbach’s alphas for emotional exhaustion, lack of personal accomplishment, and emotional distancing were 0.89, 0.84, and 0.85, respectively. Mean score of emotional exhaustion, lack of personal accomplishment, and emotional distancing were 11.94, 24.57, and 11.15, respectively. Although these results provide initial evidence for the validity of the PBI-J (e.g., acceptable model fit indexes and good internal consistencies), there were some differences between the results of the current study and those of Roskam et al. (2017). First, positive correlations between lack of parental accomplishment and emotional exhaustion or between lack of parental accomplishment and emotional distancing were weaker in the current study. Second, the score of lack of personal accomplishment was high in the current sample [24.57 in the current study vs. 9.99 (study 1) and 7.00 (study 2) in Roskam et al., 2017].

**Table 2:** Standard regression weights from CFA, mean, standard deviation, skewness, and kurtosis for each item of PBI-J.

	ED	EE	LPA	*M*	*SD*	Skewness	Kurtosis
ED1	0.495			1.23	1.83	1.41	0.80
ED2	0.653			1.22	1.60	1.20	0.43
ED3	0.559			1.91	2.01	0.72	-0.75
ED4	0.765			1.13	1.71	1.44	1.00
ED5	0.758			1.61	1.82	0.92	-0.26
ED6	0.670			1.26	1.72	1.28	0.56
ED7	0.630			1.22	1.72	1.32	0.64
ED8	0.657			1.57	1.75	0.86	-0.40
EE1		0.821		1.35	1.67	1.16	0.43
EE2		0.832		1.20	1.68	1.34	0.80
EE3		0.773		1.20	1.68	1.35	0.84
EE4		0.495		2.65	2.17	0.27	-1.33
EE5		0.814		1.60	1.76	0.97	-0.07
EE6		0.807		1.12	1.64	1.46	1.21
EE7		0.458		1.79	1.91	0.81	-0.51
EE8		0.847		1.04	1.61	1.55	1.48
LPA1			0.692	2.48	1.91	0.39	-1.02
LPA2			0.755	3.09	1.90	-0.12	-1.13
LPA3			0.743	3.48	1.87	-0.28	-1.03
LPA4			0.649	2.36	2.09	0.45	-1.16
LPA5			0.560	4.10	1.76	-0.63	-0.61
LPA6			0.672	3.06	1.99	-0.03	-1.23


*N = 1200; PBI-J, Japanese version of Parental Burnout Inventory; ED, emotional distancing; EE, emotional exhaustion; LPA, Lack of personal accomplishment. Each item’s number is matched with that of the original questionnaire by Roskam et al. (2017).*

To test whether these differences were due to the cultural differences in parental burnout specifically or burnout in general, we also conducted the same analyses for job burnout. CFA revealed that fit index of three-factor model of the JBI was nearly acceptable [CFI = 0.90, RMSEA = 0.09, χ^2^(116) = 1266.05, *p* < 0.001]. The standardized factor loadings ranged between 0.50 and 0.84. Correlations between emotional exhaustion and depersonalization, emotional exhaustion and lack of parental accomplishment, and depersonalization and lack of parental accomplishment factors were 0.92, 0.13, and 0.12, respectively. Cronbach’s alphas for emotional exhaustion, lack of personal accomplishment, and depersonalization were 0.85, 0.87, and 0.87, respectively. Mean score of emotional exhaustion, lack of personal accomplishment, and depersonalization were 11.76, 21.92, and 10.19, respectively. These results were consistent with those from the PBI-J; correlations between lack of accomplishment and emotional exhaustion or depersonalization (emotional distancing for the PBI-J) were weak, and the scores of lack of personal accomplishment were high for both parental and job burnout in Japan.

### Relationships Among PBI-J, Job Burnout, Depression, and Child-Rearing Stress

We found low to moderate positive correlations between the PBI-J total scores and depression or child-rearing stress (**Table 3**). Similar low to moderate positive correlations were found when we focused on emotional exhaustion, lack of personal accomplishment, and emotional distancing instead of the PBI-J total scores.

**Table 3:** Correlations among PBI-J (parental burnout), JBI (job burnout), depression, and child-rearing stress.

	1	2	3	4	5	6	7	8	9	10
(1) PBI-J total score	–									
(2) PBI-J EE	0.83^∗∗∗^	–								
(3) PBI-J LPA	0.42^∗∗∗^	0.02	-							
(4) PBI-J ED	0.85^∗∗∗^	0.69^∗∗∗^	0.05	–						
(5) JBI total score	**0.35^∗∗∗^**	**0.33^∗∗∗^**	**0.15^∗∗∗^**	**0.25^∗∗∗^**	–					
(6) JBI EE	0.32^∗∗∗^	**0.39^∗∗∗^**	-0.03	0.28^∗∗∗^	0.82^∗∗∗^	–				
(7) JBI LPA	0.08^∗∗^	-0.04	**0.33^∗∗∗^**	-0.07^∗^	0.53^∗∗∗^	0.06	–			
(8) JBI DP	0.37^∗∗∗^	0.38^∗∗∗^	0.02	**0.35^∗∗∗^**	0.85^∗∗∗^	0.78^∗∗∗^	0.09^∗∗^	–		
(9) Depression	**0.41^∗∗∗^**	**0.36^∗∗∗^**	**0.22^∗∗∗^**	**0.28^∗∗∗^**	0.57^∗∗∗^	0.48^∗∗∗^	0.27^∗∗∗^	0.50^∗∗∗^	–	
(10) Child-rearing stress	**0.41^∗∗∗^**	**0.49^∗∗∗^**	**0.06^∗^**	**0.28^∗∗∗^**	0.30^∗∗∗^	0.30^∗∗∗^	0.10^∗∗∗^	0.27^∗∗∗^	0.38^∗∗∗^	–
*M*	47.65	11.94	24.57	11.15	43.86	11.76	21.92	10.19	1.86	2.11
*SD*	20.93	10.76	8.54	9.88	17.19	7.25	8.03	8.25	0.57	0.64
*Range*	6–130	0–48	6–42	0–48	2–102	0–30	0–36	0–36	1–3	1–4
α	0.88	0.89	0.84	0.85	0.87	0.85	0.87	0.87	0.90	0.76


*N = 1200; PBI-J, Japanese version of Parental Burnout Inventory; EE, emotional exhaustion; LPA, lack of personal accomplishment; ED, emotional distancing; JBI,Japanese Burnout Inventory (job burnout); DP, depersonalization. ^∗∗∗^p < 0.001, ^∗∗^p < 0.01, ^∗^p < 0.05. Bold values represent correlation coefficients that are associated with the construct validity of the PBI-J.*

Overall, although there seemed to be some cultural differences in parental burnout, especially for lack of personal accomplishment, our results provided preliminary evidence for the validity of the PBI-J by showing proximity to (i.e., three-factor structure with characteristics similar to those of job burnout) and distinctiveness from job burnout (i.e., low to moderate correlations between parental and job burnout).

### Relationships Between Perfectionism, Sociodemographic Variables, and Burnout

**Table 4** indicates the correlation between perfectionism and burnout. Parental PS and CM were strongly positively correlated (*r* = 0.71, *p* < 0.001), indicating that they shared almost half of variance (i.e., *R*^2^ = 0.50). Job PS and CM were also positively correlated (*r* = 0.42, *p* < 0.001). Parental and job perfectionism total scores were positively correlated (*r* = 0.48, *p* < 0.001).

**Table 4:** Correlations between perfectionism and burnout.

	Parental perfectionism	Parental PS	Parental CM	Job perfectionism	Job PS	Job CM
PBI-J total score	0.34^∗∗∗^	0.23^∗∗∗^	0.42^∗∗∗^	0.12^∗∗∗^	-0.06^∗^	0.31^∗∗∗^
PBI-J EE	0.38^∗∗∗^	0.31^∗∗∗^	0.40^∗∗∗^	0.18^∗∗∗^	0.05	0.29^∗∗∗^
PBI-J LPA	0.02	-0.08^∗∗^	0.13^∗∗∗^	-0.13^∗∗∗^	-0.25^∗∗∗^	0.07^∗^
PBI-J ED	0.30^∗∗∗^	0.22^∗∗∗^	0.34^∗∗∗^	0.17^∗∗∗^	0.03	0.29^∗∗∗^
JBI total score	0.19^∗∗∗^	0.12^∗∗∗^	0.25^∗∗∗^	-0.02	-0.17^∗∗∗^	0.19^∗∗∗^
JBI EE	0.19^∗∗∗^	0.15^∗∗∗^	0.20^∗∗∗^	0.11^∗∗∗^	0.01	0.21^∗∗∗^
JBI LPA	-0.07^∗^	-0.13^∗∗∗^	0.01	-0.29^∗∗∗^	-0.37^∗∗∗^	-0.09^∗∗^
JBI DP	0.30^∗∗∗^	0.23^∗∗∗^	0.32^∗∗∗^	0.16^∗∗∗^	0.01	0.30^∗∗∗^
*M*	2.17	2.37	1.92	2.93	3.28	2.50
*SD*	0.96	1.05	1.04	0.90	1.08	1.05
*Range*	1–6	1–6	1–6	1–6	1-6	1-6
α	0.92	0.89	0.89	0.86	0.88	0.82


*N = 1200; PS, personal standards; CM, concern over mistakes; PBI-J, Japanese version of Parental Burnout Inventory; EE, emotional exhaustion; LPA, lack of personal accomplishment; ED, emotional distancing; JBI, Japanese Burnout Inventory (job burnout); DP, depersonalization. ^∗∗∗^p < 0.001, ^∗∗^p < 0.01, ^∗^p <0.05.*

Although both parental and job perfectionism were positively correlated with the PBI-J total scores, the association seemed to be stronger for parental perfectionism (*r* = 0.34, *p* < 0.001) than job perfectionism (*r* = 0.12, *p* < 0.001). In contrast, parental perfectionism was positively correlated with job burnout (*r* = 0.19, *p* < 0.001) whereas job perfectionism was not significantly associated with job burnout (*r* = -0.02, *p* = 0.59).

With regard to perfectionistic dimensions, parental PS was positively correlated with both parental (*r* = 0.23, *p* < 0.001) and job burnout total scores (*r* = 0.12, *p* < 0.001) whereas job PS was negatively correlated with both parental (*r* = -0.06, *p* = 0.04) and job burnout total scores (*r* = -0.17, *p* < 0.001). In contrast, both parental and job CM were positively correlated with parental and job burnout total scores.

**FIGURE 1:**
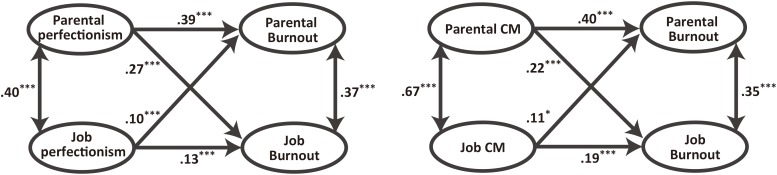
Structural equation model for the associations between perfectionism and burnout. **(Left)** Associations between overall perfectionism and burnout. **(Right)** Associations between concern over mistakes and burnout. PS, personal standards; CM, concern over mistakes. Latent variables of PS and CM, as well as indicators of PS and CM and burnout were omitted in the left figure for clarity. Indicators of CM and burnout were omitted in the right figure for clarity, ^∗∗∗^*p* < 0.001, ^∗^*p* < 0.05.

To further investigate the effect of domain specificity of perfectionism on parental and job burnout, we conducted structural equation modeling [CFI = 0.91, GFI = 0.89, RMSEA = 0.07, χ^2^(242) = 1610.44, *p* < 0.001, **Figure 1** left]. By using this analysis, we could understand the unique effect of parental and job perfectionism on job and parental burnout by controlling the influence of the other domain of perfectionism. This analysis revealed that both overall parental perfectionism and overall job perfectionism were positively associated with parental and job burnout. As expected, the positive association between parental perfectionism and parental burnout was stronger than that of job perfectionism and parental burnout [Δχ^2^(1) = 7.06, *p* = 0.008]. In addition, the positive association between parental perfectionism and parental burnout was stronger than that of job perfectionism and job burnout [Δχ^2^(1) = 18.80, *p* < 0.001].

Given that job PS was negatively correlated with both parental and job burnout, the positive association between job perfectionism and job or parental burnout may be underestimated. Thus, we also conducted structural equation modeling focusing on the parental and job CM instead of overall perfectionism to reduce the influence of job PS [CFI = 0.94, GFI = 0.94, RMSEA = 0.07, χ^2^(71) = 514.85, *p* < 0.001, **Figure 1**, right]. This analysis revealed results similar to the model of overall perfectionism. Both parental and job CM were positively associated with parental and job burnout. In addition, the positive association between parental CM and parental burnout was stronger than that of job CM and parental burnout [Δχ^2^(1) = 12.02, *p* = 0.001], and the positive association between parental CM and parental burnout was stronger than that of job CM and job burnout [Δχ^2^(1) = 22.63, *p* < 0.001].

To further investigate the associations between perfectionism dimensions and parental burnout, as well as to understand the influence of sociodemographic variables on parental burnout, we conducted multiple regression analysis (**Table 5**). This analysis revealed that although sex, age, and number of children were significantly associated with burnout, they explained little variance in parental burnout (2.8%). More specifically, younger parents reported higher parental burnout scores and having more children led to an increased parental burnout. As expected, perfectionism explained more variance in parental burnout (21.4%) than did the sociodemographic variables. More specifically, parental PS were not significantly associated with parental burnout (β = -0.05, *p* = 0.16) whereas job PS were significantly negatively associated with parental burnout (β = -0.19, *p* < 0.001). Both parental and job CM were positively associated with parental burnout (β = 0.36 and 0.22, respectively, *p* < 0.001). The results of multiple regression analysis on each PBI-J factor score was reported in Supplementary Table S1.

**Table 5:** Results of multiple regression analysis of demographic variables and perfectionism on PBI-J total scores.

	Outcome variables (β)	
Factors	PBI-J total score	JBI total score
**Step1: Sociodemographic variables**		
Gender (0 = male, 1 = female)	0.08	0.03
Age of parent	-0.10**	-0.08*
Number of children	0.06*	0.02
Having younger children (<5 years old)	-0.06	-0.06
Single parent	-0.03	-0.01
Education level	0.03	-0.01
Household income	-0.01	-0.13**
Working part-time	0.01	-0.08*
Work hours per week	-0.02	0.02
*R*^2^	2.8%***	2.7%***
**Step 2: Perfectionism**		
Parental personal standards	-0.05	0.03
Parental concern over mistakes	0.36***	0.14**
Job personal standards	-0.19***	-0.30***
Job concern over mistakes	0.22***	0.22***
*R*^2^ (*ΔR*^2^)	24.2% (21.4%***)	15.0% (12.3%***)


*N = 1097; PBI-J, Japanese version of Parental Burnout Inventory; JBI,Japanese Burnout Inventory (job burnout). ^∗∗∗^p < 0.001, ^∗∗^p < 0.01, ^∗^p < 0.05.*

The same multiple regression analysis was conducted on job burnout. This analysis revealed that although age, household income, and working part-time were significantly associated with burnout, they explained little variance in job burnout (2.7%). More specifically, younger parents reported higher job burnout scores, and having less household income and working full-time led to higher job burnout scores. Perfectionism explained more variance in job burnout (12.3%) than did sociodemographic variables. More specifically, job PS were significantly negatively associated with job burnout (β = -0.30, *p* < 0.001) whereas parental PS were not significantly associated with job burnout (β = 0.05, *p* = 0.45). Both job and parental CM were positively associated with job burnout (β = 0.22 and 0.14, respectively, *p*s < 0.004).

### Prevalence of Parental Burnout

Finally, the prevalence of parental burnout was estimated in the sample of 1,200 parents. According to the cutoff points based on scores above 67, 17.3% of the parents (21.0% mothers, 13.6% fathers) would be considered to experience burnout. According to a statistical cutoff based on 88, 4.2% of the parents (6.0% mothers, 2.3% fathers) would be considered to experience burnout.

## Discussion

In this study, we sought to understand the parental burnout in Japan by examining the validation of the PBI-J. In addition, we focused on parental perfectionism as a risk factor of parental burnout.

The present study provided preliminary evidence for validation of the PBI-J by showing proximity to and distinctiveness from job burnout. We found that the three-factor model of the PBI-J had an acceptable model fit and sufficient internal consistency, and that the PBI-J had low to moderate correlations with job burnout, parental stress, and depression. In addition, parental perfectionism, especially parental perfectionistic concerns, was positively correlated with parental burnout. Finally, the prevalence of parental burnout was estimated to be 4.2 (i.e., PBI-J scores above 88) to 17.3% (i.e., PBI-J scores above 67) in Japan.

### Similarities and Differences Between the PBI and PBI-J

The present study replicated the results from previous studies (Roskam et al., 2017; Mikolajczak et al., 2018) in several ways. First, we found the evidence suggesting that the PBI-J encompassed three-factor structures. Second, we found low to moderate correlations between parental burnout, job burnout, depression, and child-rearing stress. Third, relatively stable trait explained more variance in parental burnout than sociodemographic variables. These findings indicate that some aspects of parental burnout are common in Europe and Japan. Given that parental burnout was not simply depression or child-rearing stress, parental burnout would be one important aspect in fully understanding the parental adjustment and maladjustment.

We also found some differences from the study of Roskam et al. (2017), which can serve to invigorate future studies of parental burnout. First, total score of the PBI-J, especially for lack of personal accomplishment, was high in the current study. This finding is in line with that of a previous study showing that Japanese workers reported the highest job burnout score among other countries (e.g., Poghosyan et al., 2010). Other studies also reported that the score for lack of personal accomplishment was high in Japan (e.g., Umene-Nakano et al., 2013; Mampuya et al., 2017). Note that this result appeared to be not due to cultural differences in parental burnout specifically, but burnout in general, because the score for lack of personal accomplishment of job burnout was also high in the current study. These findings imply that similar to job burnout, Japanese parents may tend to experience parental burnout more than parents in other countries. Second, the associations between lack of personal accomplishment and emotional exhaustion or emotional distancing were weak in the current study. Previous studies have reported very weak or almost no associations between feelings of emotional exhaustion and depersonalization with personal accomplishment in Japanese workers (e.g., Tourigny et al., 2005; Poghosyan et al., 2009). Therefore, although the factor structures of the PBI-J were similar to those of the original PBI, characteristics of lack of parental accomplishment seemed to be somewhat different in Japan. One of the reasons for this outcome may be the cultural difference in parenting cognitions. Previous studies have revealed that Japanese mothers reported themselves to be less competent (Bornstein et al., 1998; Bornstein and Cote, 2004). In addition, they attributed failure or lack of success in parenting to their lack of effort, and when they succeeded in parenting, they did not attribute it to their ability (Bornstein et al., 1998; Bornstein and Cote, 2004). Such parenting cognitions may lead Japanese parents to feel less parental accomplishment.

### Perfectionism and Burnout

The results of intercorrelations between parental perfectionism and parental burnout extend previous burnout research by revealing that parental perfectionism, especially parental perfectionistic concerns, would be one of the risk factors of parental burnout. Although previous studies have indicated that perfectionism was one of the risk factors of burnout in job, education, and sport domains (e.g., Zhang et al., 2007; Stoeber and Rennert, 2008; Hill and Curran, 2016), the present study showed that the same result holds true in the parenting domain. In addition, our results of intercorrelations between perfectionism dimensions and parental burnout suggest the complex relationships among them. The results of parental perfectionistic strivings changed depending on whether perfectionistic concerns were added in the models (i.e., bivariate correlations and multiple regression analyses) while perfectionistic concerns were consistently positively associated with burnout. One of the reasons for these outcomes is that perfectionistic concerns sometimes suppress the positive aspects of perfectionistic strivings (e.g., Hill et al., 2010; Stoeber and Gaudreau, 2017; Kawamoto and Furutani, 2018). However, interpretations about the results of parental perfectionistic strivings should be made with caution because parental perfectionistic strivings and concerns were strongly positively correlated (*r* = 0.71, *p* < 0.001).

More importantly, parental perfectionistic concerns are more problematic for parental burnout than parental perfectionistic strivings. This is consistent with the view of detrimental influences of perfectionistic concerns on burnout and psychological maladjustments (e.g., Hill and Curran, 2016; Stoeber and Damian, 2016). Previous research has suggested that perfectionistic concerns are associated with increased tendencies of all-or-nothing thinking or intolerance of uncertainty, overgeneralizing negative events, and ruminating about past failures (e.g., Hewitt and Flett, 1996; Flett and Hewitt, 2002; Kawamoto and Furutani, 2018). Such cognitive characteristics may lead individuals to be vulnerable to the accrual of stress and burnout. The other possible mediators between perfectionistic concerns and burnout are controlled motivation and maladaptive coping styles (e.g., Stoeber and Janssen, 2011; Jowett et al., 2013; Stoeber and Damian, 2016). Understanding the underlying mechanism between parental perfectionistic concerns and parental burnout may help to build a comprehensive model of parental burnout.

Regarding domain specificity of perfectionism, parental perfectionism had a stronger effect on parental burnout than did job perfectionism. Our results are consistent with the argument of the importance of investigating domain-specific perfectionism on domain-specific outcome variables (e.g., Dunn et al., 2011; Stoeber and Yang, 2015). Interestingly, the positive associations between parental perfectionism or parental perfectionistic concerns and parental burnout were stronger than those of job perfectionism or job perfectionistic concerns and job burnout. A previous meta-analysis revealed that the strengths of some associations between perfectionism and burnout differed across domains (Hill and Curran, 2016). For example, they found that positive associations between perfectionistic concerns and burnout were stronger in job than in sport and education domains, presumably because perfect performance can be more ambiguous in job than in sport and education. We speculate that perfect result or performance is more ambiguous in parenting than in the job domain, which may lead to stronger associations between perfectionism and burnout in the parenting domain.

### Limitation and Future Directions

Although the current study improves our understanding of parental burnout in Japan, there are several limitations that could serve to invigorate the research on parental burnout. First, the current study utilized a cross-sectional design. Second, we only focused on working parents. To further validate the PBI-J, future studies should investigate test–retest reliability including both working and non-working parents. Third, the present study focused only on perfectionism as a risk factor of parental burnout. According to a multilevel perspective of causes on parental burnout, micro- (e.g., stable personality, personal history, and ideal parental self), meso- (e.g., sociodemographic variables or inadequate parenting practices), and macro-level factors (e.g., social conditions and expectations that increases pressure on parents) could affect parental burnout (Roskam et al., 2017). Although the current study suggested that micro-level factors could account for parental burnout more than meso-level factors, further research is needed to understand which levels of factors cause parental burnout.

## Conclusion

The present study provides preliminary evidence of the validity of the PBI-J. In addition, parental perfectionism, especially perfectionistic concerns, seems to be one of the risk factors of parental burnout. Some differences between the current study and the study by Roskam et al. (2017) warrant a need for investigating cultural commonalities and specificities of parental burnout. Cross-cultural investigations of parental burnout would invigorate the development of theories and models as well as help to see a complete picture of parental burnout, which we believe will lead to a reduced prevalence of parental burnout.

## Author Contributions

All authors listed have made a substantial, direct and intellectual contribution to the work, and approved it for publication.

## Conflict of Interest Statement

The authors declare that the research was conducted in the absence of any commercial or financial relationships that could be construed as a potential conflict of interest.
