# Supplementary material for: Preliminary Validation of Japanese Version of the Parental Burnout Inventory and Its Relationship With Perfectionism
Source: Front Psychol. 2018 Jun 20;9:970. doi: 10.3389/fpsyg.2018.00970 (PMC6019475; doi:10.3389/fpsyg.2018.00970)
Supplement: Supplementary file 1 [file Table_1.DOCX]

**Table S1. Results of multiple regression analysis for demographic variables and perfectionism on PBI-J factor scores**

|  | Outcome variables (β) | | |
| --- | --- | --- | --- |
| Factors | PBI-J EE | PBI-J ED | PBI-J LPA |
| **Step1: Sociodemographic variables** |  |  |  |
| Gender (0 = male, 1 = female) | 0.17^***^ | 0.10^*^ | -0.12^**^ |
| Age of parent | -0.14^***^ | -0.04 | -0.04 |
| Number of children | 0.04 | 0.05 | 0.04 |
| Having younger children (< 5 years old) | 0.04 | -0.08^*^ | -0.11^**^ |
| Single parent | -0.03 | -0.02 | -0.01 |
| Education level | 0.06 | 0.06^*^ | -0.07^*^ |
| Household income | -0.01 | 0.02 | -0.06 |
| Working part-time | 0.01 | 0.03 | -0.03 |
| Work hours per week | -0.02 | -0.03 | 0.01 |
| *R^2^* | 7.9%^***^ | 2.6%^**^ | 3.3%^***^ |
| **Step 2: Perfectionism** |  |  |  |
| Parental personal standards | 0.07 | -0.01 | -0.23^***^ |
| Parental concern over mistakes | 0.27^***^ | 0.24^***^ | 0.28^***^ |
| Job personal standards | -0.06 | -0.08^*^ | -0.28^***^ |
| Job concern over mistakes | 0.13^***^ | 0.21^***^ | 0.13^*^ |
| *R^2^* (*ΔR^2^*) | 23.2% (15.2%^***^) | 16.4% (13.8%^***^) | 17.3% (13.9%^***^) |

Note: *N* = 1097, PBI-J: Japanese version of Parental Burnout Inventory, EE: emotional exhaustion, LPA: lack of personal accomplishment, ED: emotional distancing. ^***^*p* < .001, ^**^*p* < .01, ^*^*p* < .05.
